# Shexiang-Wulong Pills Attenuate Rheumatoid Arthritis by Alleviating Inflammation in a Mouse Model of Collagen-Induced Arthritis

**DOI:** 10.1155/2019/5308405

**Published:** 2019-02-11

**Authors:** Zhihui Zhang, Ying Cao, Qiang Yuan, Aiguo Zhang, Kaiqi Zhang, Zhiwen Wang

**Affiliations:** ^1^College of Traditional Chinese Medicine, North China University of Science and Technology, No. 21 Bohai Road, Caofeidian Xincheng, Tangshan 063210, Hebei, China; ^2^Affiliated Hospital of North China University of Science and Technology, No. 73 Jianshe Road, Lubei District, Tangshan 063210, Hebei, China

## Abstract

Shexiang-Wulong Pill (SWP) is derived from “*Moschus* Yuan,” first formulated during the Song Dynasty for the treatment of joint pain. The aim of this study was to evaluate the therapeutic effect of SWP in a mouse model of collagen-induced arthritis (CIA). Forty-five DBA/1 mice were randomly divided into control group, model group, and SWP-treated group. SWP was administered by oral gavage for 22 days after the booster immunization. The clinical arthritic scores and joint histopathology, including synovial hyperplasia and hypoxic regions, cartilage erosion, and bone destruction, were evaluated. Microcomputed tomography (micro-CT) was used to assess microstructural changes in the bone. Serum levels of TNF-a, IL-6, and IFN-*γ* were measured by enzyme-linked immunosorbent assay (ELISA). The results showed a statistically significant improvement in joint pathological changes in the SWP-treated group. Imaging assessment confirmed that SWP protected the bone tissue from CIA-induced erosion and increased the bone density. In addition, the serum levels of TNF-a, IL-6, and IFN-*γ* in SWP-treated mice were significantly lower than those in the model group (*P*<0.05). Taken together, Shexiang-Wulong Pill can effectively alleviate joint swelling in CIA mice, inhibit synovial tissue hyperplasia, reduce inflammatory cell infiltration, and delay bone destruction. These results indicate that Shexiang-Wulong Pills could be an efficient medication for the treatment of RA.

## 1. Introduction

Rheumatoid arthritis (RA) is a systemic autoimmune disease characterized by synoviocyte hyperplasia, inflammatory cell infiltration, synovial pannus formation, and the destruction of cartilage and bone [1]. Progressive joint damage results in swelling and pain and limits joint movement. The global prevalence of RA is 0.5-1% [2]. After the onset of the disease, patients often suffer from lifelong illness, which seriously reduces the life quality and also causes a huge economic burden on the family and society.

Since the pathogenesis of RA has not been completely elucidated, RA's therapeutic strategy is concentrated on multiple targets. Conventional drugs such as nonsteroidal anti-inflammatory drugs (NSAIDs), corticosteroids, and disease-modifying antirheumatic drugs (DMARDs) may relieve the symptoms of the disease but are associated with adverse effects like kidney and liver injury, bone loss, and immunosuppression during long-term use. Biological agents such as tumor necrosis factor (TNF) inhibitors are considered to be more effective than former drugs [3]. However, approximately 50% of patients discontinue them over the course of the first five years as a result of inefficacy or adverse events [4]. In recent years, accumulating evidence indicates that Chinese medicine may become a novel strategy for the treatment of RA. For example,* Tripterygium wilfordii* can reduce the expression of proinflammatory cytokines and matrix metalloproteinases (MMPs) and inhibit the proliferation of synovial fibroblasts [5]. A systematic review with meta-analysis showed that* Tripterygium wilfordii* extract monotherapy or combination with DMARDs significantly improved the RA symptoms and had an acceptable safety profile [6].* Radix Paeoniae Alba* reduced the levels of IL-1*β* and TNF-*α* and relieved inflammation and bone erosion in a rat model of CIA [7]. A recent meta-analysis showed that, compared to conventional drugs, traditional Chinese medicine (TCM) formulations significantly increased bone density and reduced the serum levels of MMP-3 in arthritic patients [8].

Shexiang-Wulong Pill (SWP) is derived from* Moschus Yuan* that was first formulated by Shuwei Xu during the Song Dynasty. For more than nine hundred years,* Moschus Yuan* has been the basic prescription for treating joint pains among TCM practitioners. SWP is an orally administered pill composed of* Moschus*,* Aconiti Radix*,* Scorpio, Pheretima,* and* Sojae semen nigrum* and is only produced by the Affiliated Hospital of North China University of Science and Technology to treat patients with RA and degenerative osteoarthritis. Our previous clinical study showed that SWP significantly improved the tender joint count, swollen joint count, morning stiffness, hand grip strength, and visual analog pain scores in RA patients compared to the Total Glucosides of Paeonia capsules [9]. SWP also reduced synovial hypoxia and edema in rabbits with papain-induced knee osteoarthritis [10] and accelerated the union of femoral fracture in ovariectomized rats [11].

In this study, we evaluated the efficacy of SWP in a mouse model of CIA, which mimics the clinical symptoms, pathological features of synovitis, imaging characteristics, and immunological indicators of human RA [12]. Synovitis and immune cell infiltration are the primary pathological manifestations of joint lesions in RA. In addition, synovial hypoxia can induce inflammation and angiogenesis, which is vital for pannus formation [13, 14]. Therefore, we assessed the efficacy of SWP in the CIA model on the basis of symptomatic scores, pathological changes, hypoxia, imaging evaluation, and the levels of inflammation.

## 2. Materials and Methods

### 2.1. Animals

Male DBA/1 mice (weighing 18-20 g) were purchased from Beijing Huakang Biotechnology Co. Ltd., license number SCXK (Beijing) 2014-0004. All animal studies were performed with the protocol approved by the Animal Care Welfare Committee of North China University of Science and Technology.

### 2.2. Preparation of Collagen Type II (CII) Emulsion

Immunization grade bovine type II collagen (Chondrex Inc., Redmond, USA) was dissolved in 0.1M glacial acetic acid (Jindongtianzheng Precision Chemical Reagent Factory, Tianjin, China) at the concentration of 2 mg/ml and stirred overnight at 4°C. It was then mixed with an equal amount of Freund's complete adjuvant (Sigma-Aldrich, St. Louis, USA) and emulsified with a homogenizer on ice. When the emulsion drop floats completely on the surface of the water without spreading out, it indicates complete emulsification [15, 16].

### 2.3. Establishment of CIA Model and Treatment Protocol

After acclimatizing for 1 week, mice (n =30), except the control group, were intradermally injected with 0.1 ml CII emulsion at the base of the tail for the first immunization and the booster dose was given on the 21st day. These were recorded as Day1 and Day21, respectively [12, 13]. The control mice (n =15) were injected with the same volume of 0.9% saline. SWPs (Approval of Clinical Study of Pharmaceutical Preparation of Medical Institution No. Z20051581, Patent No. CN101574413) were obtained from Affiliated Hospital of North China University of Science and Technology, which were dissolved in distilled water at the concentration of 50 mg/ml and administered gastrointestinally. After the booster CII dose, the mice were randomized into the SWP-treated and untreated model groups (n=15 each) and accordingly administered SWP or saline via the gastrointestinal route till day 43; the control group mice also received saline for the same duration.

The body weight of all the mice was recorded once every 7 days and the severity of paw arthritis was scored every 3 days from Day 21, with the maximum score of 4 points per foot. The scoring criteria were as follows: Grade 0: normal, with no redness; Grade 1: mild, with skin redness or slight redness and swelling in 1-2 toe joints; Grade 2: moderate, with slight redness and swelling in the feet, foot pads, or ankles; Grade 3: serious, with considerable redness and swelling in the feet, toes, and joints; and Grade 4: severe, with highly swollen and red feet, toes, and joints, in addition to stiffness and deformity. The total score of the four feet was calculated as the arthritis score for each mouse. Arthritis was defined with at least 1 foot with clinical score ≧ 2. On Day 43, all the mice were sacrificed and the knee joints were resected for further analysis.

### 2.4. Histological Assessment

The knee joints were fixed in 4% paraformaldehyde for at least 48 h and decalcified for 4 weeks in 10% ethylene-diamine-tetra acetic acid (EDTA). Thereafter, the samples were routinely dehydrated, paraffin-embedded, and cut into 5 *μ*m sections. The histological changes in knee joints of mice were observed by hematoxylin and eosin staining (H&E, Baso Diagnostics Inc., Zhuhai, China) and Safranin-O staining (Beijing Leagene Biotechnology Co. Ltd., Beijing, China) according to the manufacturer's instructions. Arthritis was evaluated using the CellSens Dimension software and cartilage damage was scored by Mankin's criteria [17].

### 2.5. Hypoxia Probe Injection and Detection by Immunohistochemical (IHC) Staining

The hypoxia probe pimonidazole (Hypoxyprobe Inc., Burlington, USA) was intraperitoneally injected at the dose of 60 mg/kg. The mice were sacrificed, and the knee joints were resected and processed as described in the preceding section. After decalcification, the deparaffinized sections were incubated with primary antibodies overnight at 4°C. The subsequent steps were performed in accordance with the instructions of the Secondary Antibody Kit (PV-6000, Zhongshan Jinqiao, China).

### 2.6. Microstructure Analysis

Knee joints were fixed in 4% paraformaldehyde for 48 h and then washed with phosphate-buffered saline (PBS) and soaked in 75% ethanol. The SkyScan-1176 micro-CT (Bruker micro- CT, Belgium) system was used to scan the microstructural changes of knee joints. Micro-CT was performed on a 1 mm region of metaphyseal spongiosa in the distal femur, 0.5mm above (femur) the growth plate, to evaluate the trabecular bone. The images were reconstructed and realigned in 3D using NR econ software, version 1.6. The bone parameters, including bone mineral density (BMD), bone volume fraction (BV/TV), trabecular bone thickness (Tb.Th), trabecular bone number (Tb.N), trabecular bone separation (Tb.Sp), trabecular pattern factor (TPF), and structure model index (SMI), were evaluated using the CT software, version 1.13.

### 2.7. Concentration of Cytokines in Serum

The blood was collected through eyeball removal after anesthetization, centrifuging at 4°C for 15 min at 3000 r/min to separate the serum. The concentration of TNF-*α*, IL-6, and IFN-*γ* was measured using Enzyme-linked immunosorbent assay (ELISA) kit (Jiangsu MeiBiao Biological Technology Co., Ltd., Jiangsu, China) in strict accordance with the manufacturer's instructions.

### 2.8. Statistical Analysis

SPSS 22.0 was used for data analysis. All data are expressed as mean ± standard deviation, and one-way ANOVA was used for comparison among multiple groups. LSD was used between the two groups when the homogeneity of variance was satisfied, and Dunnett's T3 was used when the homogeneity of variance is not satisfied. P<0.05 was considered statistically significant.

## 3. Results

### 3.1. SWP Attenuated the Severity of Arthritis and Body Weight Loss in CIA Mice

**Figure 1:**
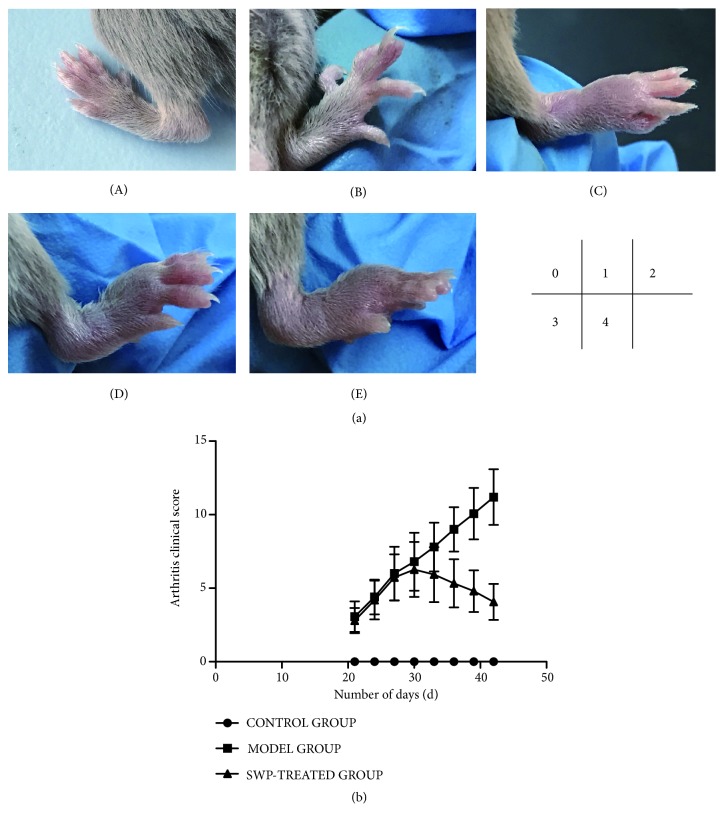
Effect of SWP on the clinical score of each group. (a) Evaluation criteria of CIA clinical arthritis scores. (A)-0, (B)-2, (C)-3, (D)-4, and (E)-5. (b) Comparison of arthritis clinical scores among all groups.

According to the evaluation criteria (Figure 1(a)), the clinical arthritis score in the CIA model group was significantly higher than that of the control group from Day 21 onwards (*P*<0.01). In some modelled mice, arthritic symptoms like joint swelling and loss of appetite were manifested prior to the CII booster, while all mice showed the typical symptoms by Day 30 and peaked on Day 33. The arthritis clinical score in SWP-treated group was significantly less than that of the model group From Day 33 to Day 42 (*P*<0.05)(Figure 1(b)).

**Figure 2:**
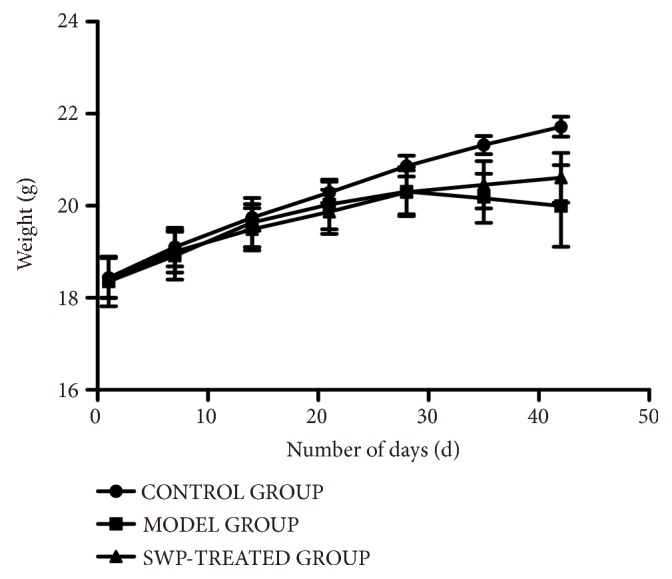
Graph showing the alterations in body weight of the mice in each group.

As shown in Figure 2, the body weight showed an upward trend with no obvious difference among three groups before Day 21 (*P*>0.05) and continued in the untreated control group till Day 42. However, CIA mice had body weight loss from Day 28 to Day 42, suggesting that the weight loss in the treatment group may be associated with inflammation. At Day 42, compared with the control group, the weight of CIA mice decreased significantly (*P*<0.01). In contrast, the SWP-treated mice showed no obvious weight loss compared to the control group, which was significantly higher than that of the model group (*P*<0.05) after the 42^nd^ day. Taken together, the CII-induced arthritic model was established successfully, and SWP showed significant therapeutic effects against the typical symptoms.

### 3.2. SWP Attenuated the Pathological Changes of Knee Joints in CIA Mice

**Figure 3:**
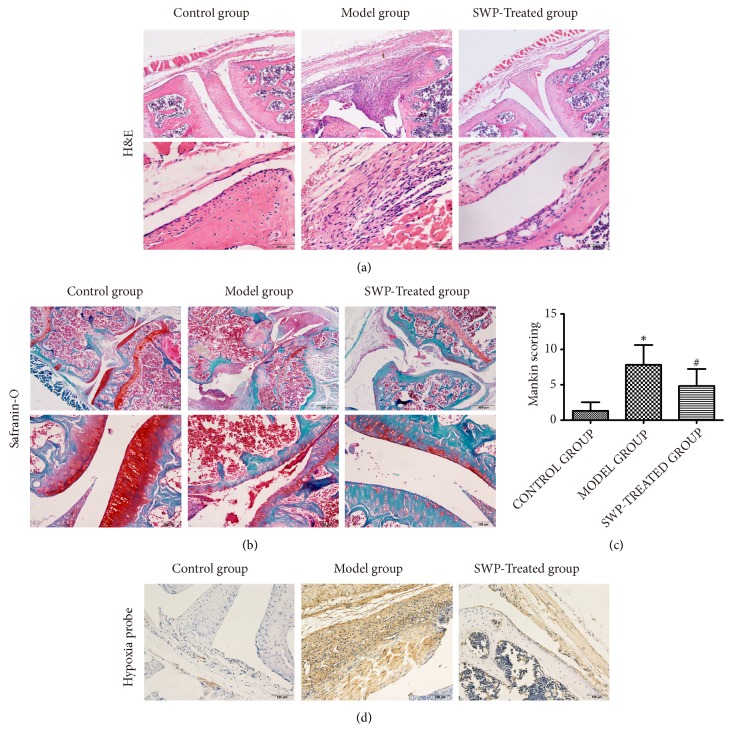
Effect of SWP on the histopathological changes in the arthritic knee joints. (a) H&E staining of knee joints in each group. (b) Safranin-O staining of knee joints in each group. (c) Comparison of Mankin scores of different groups. *∗P*<0.05, compared to control group. ^#^*P*<0.05, compared to model group. (d) Hypoxia assessments of the knee synovial membrane in each group.

Histological examination showed that the synovial membrane of knee joints from the control mice had regular cellular arrangement, without any synoviocyte hyperplasia, inflammatory cell infiltration, or pannus formation, in addition to a smooth cartilage surface and intact bone. In contrast, the arthritic mice had severe inflammation, synoviocyte hyperplasia, and extensive cartilage and bone erosion, and these symptoms were significantly reduced in the SWP-treated mice (Figure 3(a)). Safranin-O staining (Figure 3(b)) and Mankin scores (Figure 3(c)) indicated a thinner cartilage layer with a rough surface, decreased chondrocyte counts, and multiple tide lines and pannus in the arthritic joints. SWP treatment significantly lowered the mean Mankin score and restored the cartilage in the model group (*P*<0.05). Hypoxia probe staining was performed and assessed by immunohistochemistry to detect hypoxic regions. We found that the area of hypoxia region was significantly decreased in the joint of SWP-treated mice than that in model group. (Figure 3(d)).

### 3.3. SWP Attenuated Bone Destruction in CIA Mice

**Figure 4:**
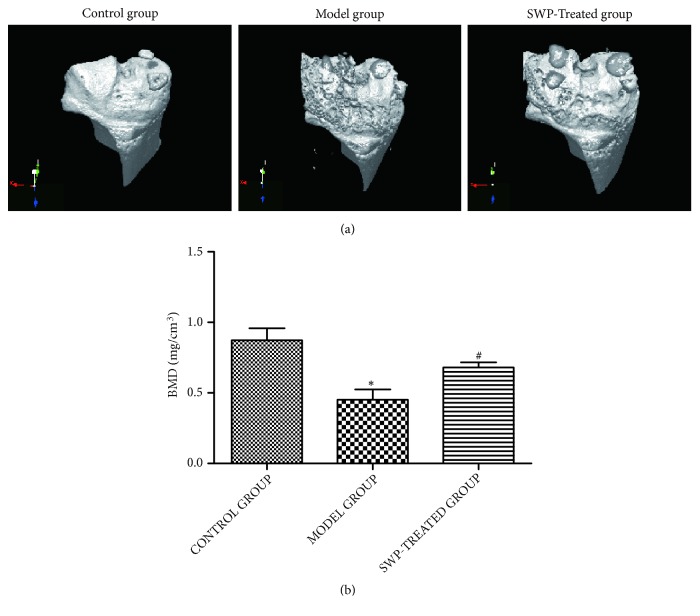
Effect of SWP on the bone mineral density (BMD) of each group (a) Three-dimensional reconstruction of the knee joint for each group. (b) BMD values of each group. *∗P*<0.05 compared to control group, ^#^*P*<0.05 compared to model group.

Three-dimensional micro-CT revealed an intact knee joint structure with a smooth bone surface in the control group, whereas that of the arthritic mice was severely eroded, with signs of osteoporosis (Figure 4(a)). SWP treatment markedly reduced the extent of joint damage. Compared to the control mice, the BV/TV, Tb.Th, Tb.N, and BMD were significantly reduced (*P* < 0.05), and Tb.Sp, TPF, and SMI were significantly increased in the CIA mice (*P* < 0.05). These parameters were significantly improved after SWP treatment (Table 1, Figure 4(b)), which indicates a protective role of SWP on inflammation mediated bone destruction.

### 3.4. SWP Reduced the Serum Levels of TNF-a, IL-6, and IFN-*γ* in CIA Mice

Compared to the control group, the serum levels of TNF-a, IL-6, and IFN-*γ* were significantly increased in the arthritic mice and markedly restored by SWP treatment (P<0.05; Table 2). This indicates that CII-induced inflammation likely mediated the pathological changes seen in the arthritic mice, and SWP alleviated the inflammatory response.

## 4. Discussion

RA is a chronic progressive autoimmune disease characterized by joint swelling, pain, and deformity. It manifests in the early stages as synovitis, eventually leading to cartilage erosion and bone destruction [18]. The current therapeutic agents against RA only provide short-term relief from the symptoms and are associated with several adverse effects. We simulated the arthritic symptoms in DBA/1 mice by injecting them with an emulsification of bovine CII and Freund's complete adjuvant and treated the CIA mice with SWP, a herbal formulation used to treat joint pains in China. The initial symptoms of joint swelling started appearing 3 weeks after the primary injection and gradually intensified to the entire forefeet, with a concomitant increase in the clinical arthritic scores and histopathological changes. SWP treatment significantly reduced the joint swelling and histological scores in the CIA mice. The knee joints of the SWP-treated mice showed a thinner synovium, decreased inflammatory cell infiltration, reduced cartilage erosion, and a reduction in synovial hyperplasia, which in turn decreased the anoxic area of the synovium and angiogenesis.

To further evaluate the therapeutic efficacy of SWP, a three-dimensional image of the knee was reconstructed using micro-CT to assess the microstructural changes in the knee joint, in terms of several indicators of bone quality, including BV/TV or BVF, Tb.N, and Tb.Th [19]. BVF is an indicator of the cancellous bone microstructure, and a reduction in BVF represents bone loss. An increase in SMI from 0 to 3 indicates a transition from plate-like to the rod-like trabecular bone during the onset of osteoporosis [20]. Tb.N, Tb.Th, and Tb.Sp are microarchitectural parameters which are also used to evaluate osteoporosis. The CIA mice in our study showed bone destruction and osteoporosis, while SWP protected the knee joints from bone loss. In a previous study, we found that SWP increased the levels of serum calcium, phosphorus, and alkaline phosphatase, as well as the proliferation of osteoblasts, to increase the bone density in a rabbit model of bone fracture [21, 22].

We also detected severe weight loss in the CIA mice, similar to rheumatoid cachexia or the loss of body cell mass (BCM), which could be attributed to mental fatigue, poor appetite, and low physical activity after the onset of symptoms. Studies have reported an average loss of 13-15% BCM among RA patients, and even a 5% loss can increase the susceptibility to infections [23]. The mice treated with SWP gradually gained weight compared to the model group, indicating that orally administered SWP effectively controlled the symptoms of the disease.

Chronic synovitis is the pathological basis of RA. The autoimmune reaction triggers an inflammatory response which increases vascular permeability and further promotes immune cell infiltration that produces large amounts of inflammatory cytokines. This vicious cycle causes synovial hyperplasia and pannus formation, which lead to cartilage erosion and eventually cause bone destruction [24]. The proinflammatory cytokines TNF-a, IL-6, and IFN-*γ* play a synergistic role in the progression of RA, causing synovial inflammation, cartilage damage, and bone destruction [25]. TNF-*α* induces not only IL-6 and IFN-*γ* production [26] but also osteoclast recruitment, which contributes to bone resorption [27]. Previous studies have shown that IL-6 is the most predominant cytokine in sera of RA patients and is correlated with disease activity [28], and blocking it significantly alleviated the symptoms of RA [29]. IL-6 also increases the expression of adhesion molecules and recruits leukocytes to the injured synovial membranes [30]. Furthermore, IL-6 binds to its specific receptor on the osteoclasts, which is a crucial step in chronic arthritis [31]. IFN-*γ* is also a marker for RA [32] and activates the fibroblast-like synoviocytes (FLS), which express major histocompatibility complex II (MHCII) and therefore induce T cells proliferation [33]. SWP markedly reduced the circulating levels of TNF-a, IL-6, and IFN-*γ*, which could be the basis of the remission of symptoms seen in the CIA mice. This is consistent with our previous study wherein SWP-treated arthritic rats showed a significant reduction in the synovial levels of TNF-a and IL-6 [34]. In a study on RA patients, SWP treatment significantly decreased serum levels of IL-18 and Fractalkine (FKN)/CX3CL1 [9]. Soluble FKN induces activated monocytes to secrete IL-6 [35], while IL-18 directly stimulates macrophages to secrete TNF-*α*, IL-6, and IFN-*γ* in combination with IL-12. Another study showed that the serum levels of IL-6 were significantly reduced in CIA mice after neutralization of IL-18 [36]. Therefore, SWP may decrease the secretion of proinflammatory cytokines by inhibiting IL-18 and FKN/CX3CL1.

Several TCM formulations alleviated the symptoms of RA in CIA models by decreasing inflammation. For example, Xianfanghuomingyin modulates the differentiation of Th1, Th2, and Th17 cells and activates the Treg cells [37], Bi-Qi capsule decreases serum levels of TNF-*α* and IL-18 [38], and Hei-Gu-Teng Zhuifenghuoluo granules reduced TNF-*α*, IL-1*β*, and IL-6 in the sera and synovia of CIA mice [39]. High levels of the inflammatory cytokines TNF-*α*, IL-6, and IL-17 have been detected in the synovial fluid of RA patients and are directly correlated with cartilage and bone destruction. TNF-*α* and IL-1 degrade the cartilage by inducing chondrocytes to secrete metalloproteases. In addition, all three cytokines induce the expression of receptor activator of NF-*κ*B (RANK) ligand (RANKL), which is known to promote osteoclastogenesis. Osteoprotegerin (OPG) is a decoy for RANKL and can competitively bind to RANK. The RANKL/OPG ratio is therefore a critical regulator of osteoclast differentiation and function [40, 41]. Celastrol, a Chinese herb-derived compound, attenuated inflammation and bone damage in a rat model of adjuvant-induced arthritis rats by suppressing IL-17 and IL-6 and decreasing the RANKL/OPG ratio [42, 43]. Therefore, TCM formulations including SWP can be considered as complementary or alternative medications for treating RA.

## 5. Conclusions

Shexiang-Wulong Pills alleviated synovial hyperplasia and hypoxia, cartilage erosion, and bone destruction caused by CIA, possibly due to the reduction in the proinflammatory cytokines. SWP is a potential therapeutic alternative for RA that warrants further clinical studies.

## Figures and Tables

**Table 1:** Comparison of Micro-CT parameters of knee joints in each group.

Parameter	Control Group	Model Group	SWP-Treated Group
BV/TV	44.822±5.279	13.313±5.627^*∗*^	31.679±2.976^#^
Tb.Th	0.043±0.003	0.038±0.003^*∗*^	0.045±0.001^#^
Tb.Sp	0.097±0.014	0.171±0.021^*∗*^	0.117±0.004^#^
Tb.N	10.412±0.885	3.313±1.085^*∗*^	7.034±0.681^#^
TPF	-7.466±4.057	26.091±9.914^*∗*^	6.465±4.988^#^
SMI	-0.633±0.383	1.647±0.392^*∗*^	0.503±0.381^#^

*∗P*<0.05, when compared with control group, ^#^*P*<0.05, when compared with model group.

**Table 2:** Effect of SWP on serum TNF-a, IL-6, and IFN-*γ* for each group.

Group	N	TNF-a	IL-6	IFN-*γ*
Control group (pg/ml)	8	450.45±18.85	73.38±6.87	505.56±75.11
Model group (pg/ml)	8	700.80±113.97^*∗*^	117.10±14.59^*∗*^	654.02±53.07^*∗*^
SWP-Treated group (pg/ml)	9	535.20±29.29^#^	99.75±9.47^#^	587.90±57.99^#^

*∗P*<0.05 when compared with control group, ^#^*P*<0.05 when compared with model group.

## Data Availability

The data used to support the findings of this study are available from the corresponding author upon request.
